# Supplementary material for: Synthesis, Characterization, and Preliminary In Vitro Cytotoxic Evaluation of a Series of 2-Substituted Benzo [d] [1,3] Azoles
Source: Molecules. 2021 May 8;26(9):2780. doi: 10.3390/molecules26092780 (PMC8125891; doi:10.3390/molecules26092780)
Supplement: Supplementary file 1 [file molecules-26-02780-s001.zip › molecules-1131434-supplementary.pdf]

Synthesis, Characterization and Preliminary in vitro Cytotoxic Evaluation of a Series of 2-Substituted Benzo[d][1,3]azoles

Ozvaldo Linares-Anaya,[1] Alcives Avila-Sorrosa,[1,*] Francisco Díaz-Cedillo,[1] Luis A. Gil-Ruiz,[1,2] José Correa-Basurto,[2] Domingo Salazar-Mendoza,[3] Adrian L. Orjuela,[4] Jorge Alí-Torres,[4] María Teresa Ramírez-Apan[5] and David Morales-Morales[5]

1 Instituto Politécnico Nacional, Escuela Nacional de Ciencias Biológicas, Departamento de Química Orgánica, Carpio y Plan de Ayala S/N, Colonia Santo Tomás, 11340, Ciudad de México, México.
2 Laboratorio de Diseño y Desarrollo de Nuevos Fármacos e Innovación Biotecnológica, Escuela Superior de Medicina, Instituto Politécnico Nacional, Ciudad de México, 11340, México.
3 Universidad Tecnológica de la Mixteca, Carretera a Acatlima 2.5 km, 69000, Huajuapan de León, Oaxaca, México.
4 Departamento de Química, Universidad Nacional de Colombia-Sede Bogotá, 111321, Colombia.
5 Instituto de Química, Universidad Nacional Autónoma de México, Circuito Exterior, Ciudad Universitaria, Ciudad de México, C.P. 04510, México.
* Correspondence: aavilas@ipn.mx; Tel.: +52-555-729-6000, ext. 62414 (A.A.-S.)

Contents

NMR spectra for 2-substituted benzo[*d*][1,3]azoles derivatives (**BTA-1, BZM-2, BOX-3, BTA-4, BZM-5 and BOX-6**).

Figure S1: ^{1}H NMR (300 MHz, $CDCl_3$) Spectrum for **BTA-1**.

Figure S2: ^{13}C{^{1}H} NMR (75 MHz, $CDCl_3$), Spectrum for **BTA-1**.

Figure S3: Mass spectrometry (EI) for **BTA-1.**

Figure S4: ^{1}H NMR (300 MHz, $CDCl_3$) Spectrum for **BZM-2**.

Figure S5: ^{13}C{^{1}H} NMR (75 MHz, $CDCl_3$), Spectrum for **BZM-2**.

Figure S6: Mass spectrometry (DART$^+$) for **BZM-2.**

Figure S7: ^{1}H NMR (300 MHz, $CDCl_3$) Spectrum for **BOX-3**.

Figure S8: ^{13}C{^{1}H} NMR (75 MHz, $CDCl_3$), Spectrum for **BOX-3**.

Figure S9: Mass spectrometry (EI) for **BOX-3.**

Figure S10: ^{1}H NMR (300 MHz, $CDCl_3$) Spectrum for **BTA-4**.

Figure S11: ^{13}C{^{1}H} NMR (75 MHz, $CDCl_3$) Spectrum for **BTA-4**.

Figure S12: Mass spectrometry (DART$^+$) for **BTA-4.**

Figure S13: ^{1}H NMR (300 MHz, $CDCl_3$) Spectrum for **BZM-5**.

Figure S14: ^{13}C{^{1}H} NMR (75 MHz, $CDCl_3$) Spectrum for **BZM-5**.

Figure S15: Mass spectrometry (DART$^+$) for **BZM-5.**

Figure S16: ^{1}H NMR (300 MHz, $CDCl_3$) Spectrum for **BOX-6**.

Figure S17: ^{13}C{^{1}H} NMR (75 MHz, $CDCl_3$) Spectrum for **BOX-6**.

Figure S18: Mass spectrometry (DART$^+$) for **BOX-6.**

Table S1. Docking results of receptor EGFR with crystalized ligand as reference and tamoxifen.

Table S2. Docking result of receptor Erα with crystalized ligand as reference and tamoxifen.

Table S3. Docking result of receptor Pr with crystalized ligand as reference and Tamoxifen.

Table S4. Docking result of receptor mTOR with crystalized ligand as reference and Tamoxifen.

Figure S19. Redocking of crystalized ligand in A) EGFR receptor B) Erα receptor C) mTOR receptor D) Pr Receptor.

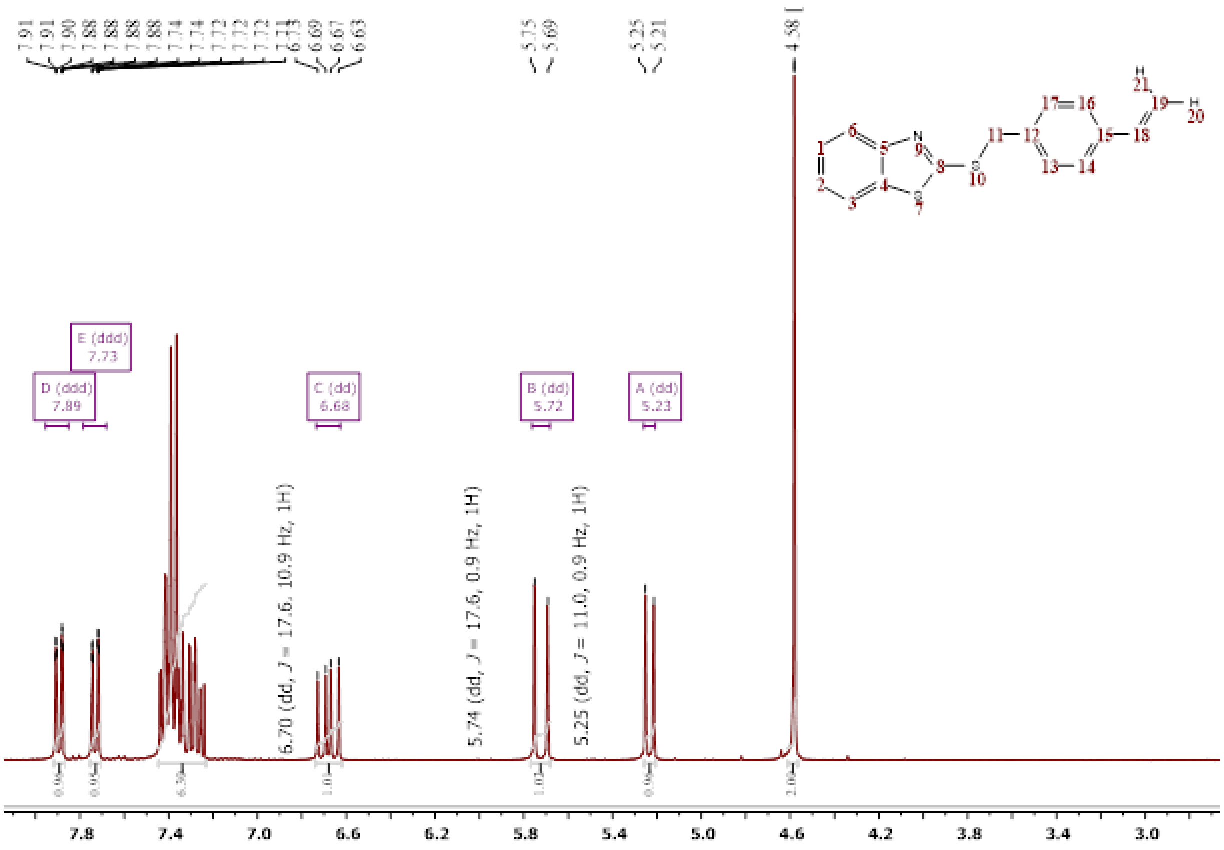

Figure S1. ^{1}H NMR (300 MHz, $CDCl_3$) Spectrum for **BTA-1**.

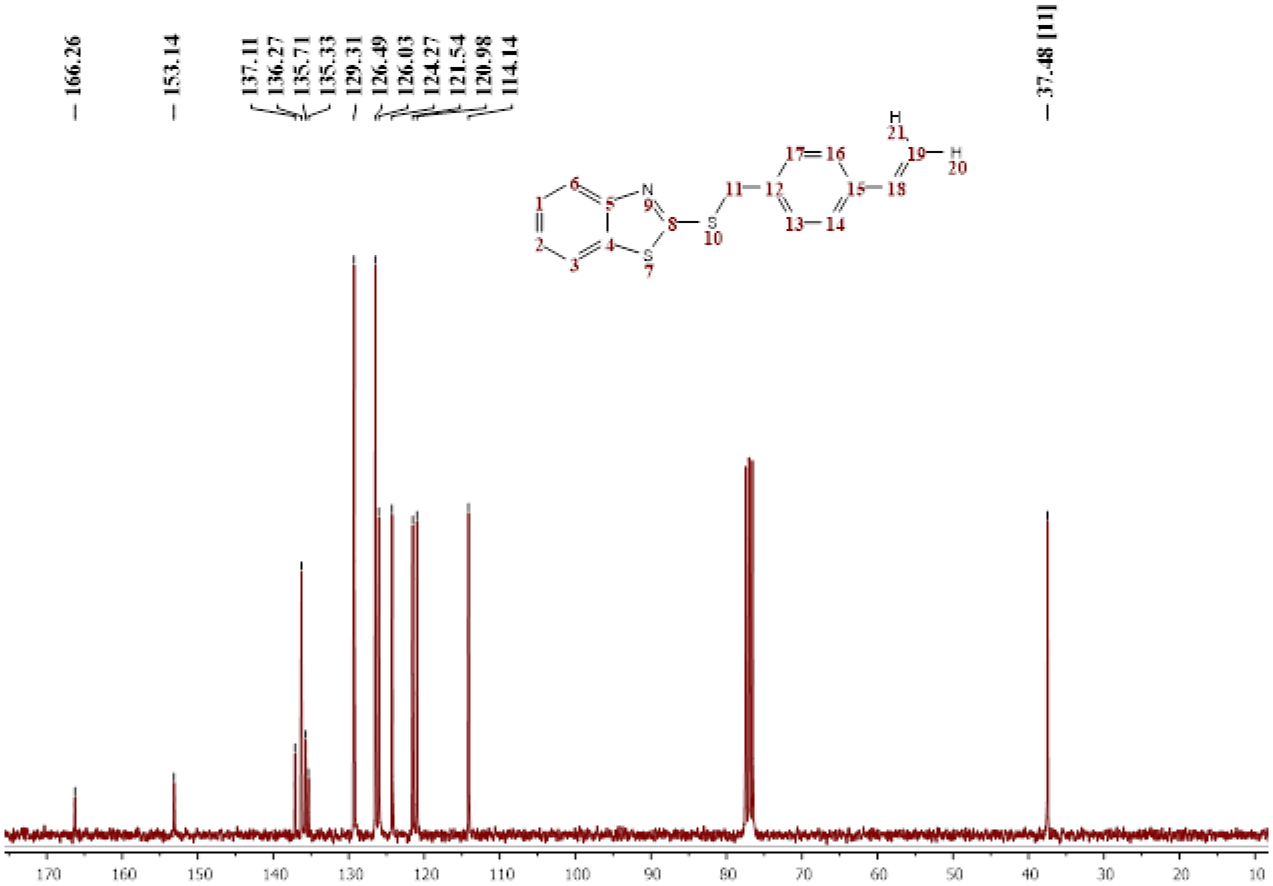

Figure S2. $^{13}C\{^{1}H\}$ NMR (75 MHz, $CDCl_3$) Spectrum for **BTA-1**..

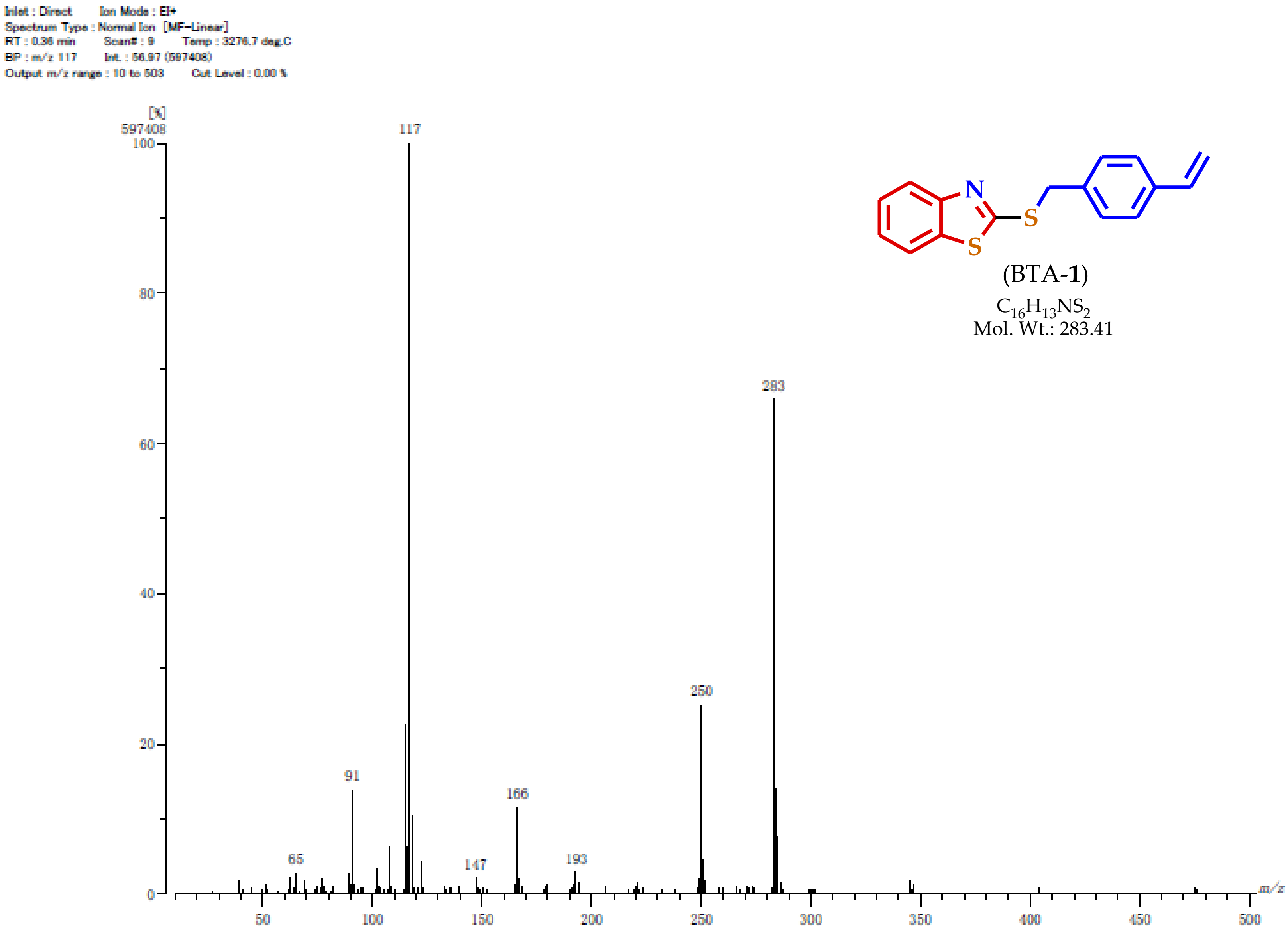

Figure S3. Mass spectrometry (EI) for **BTA-1**

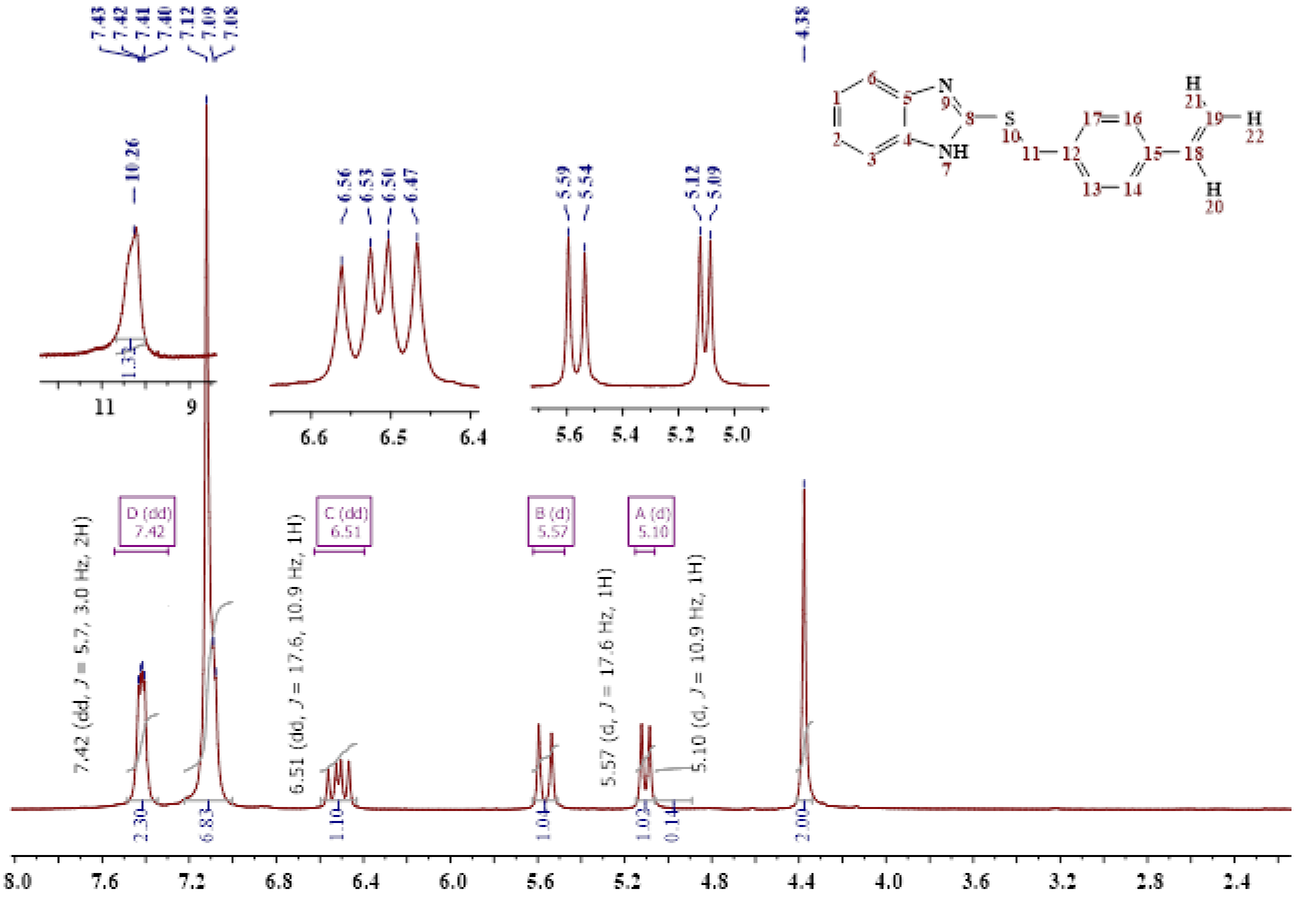

Figure S4. ^{1}H NMR (300 MHz, $CDCl_3$) Spectrum for **BZM-2**.

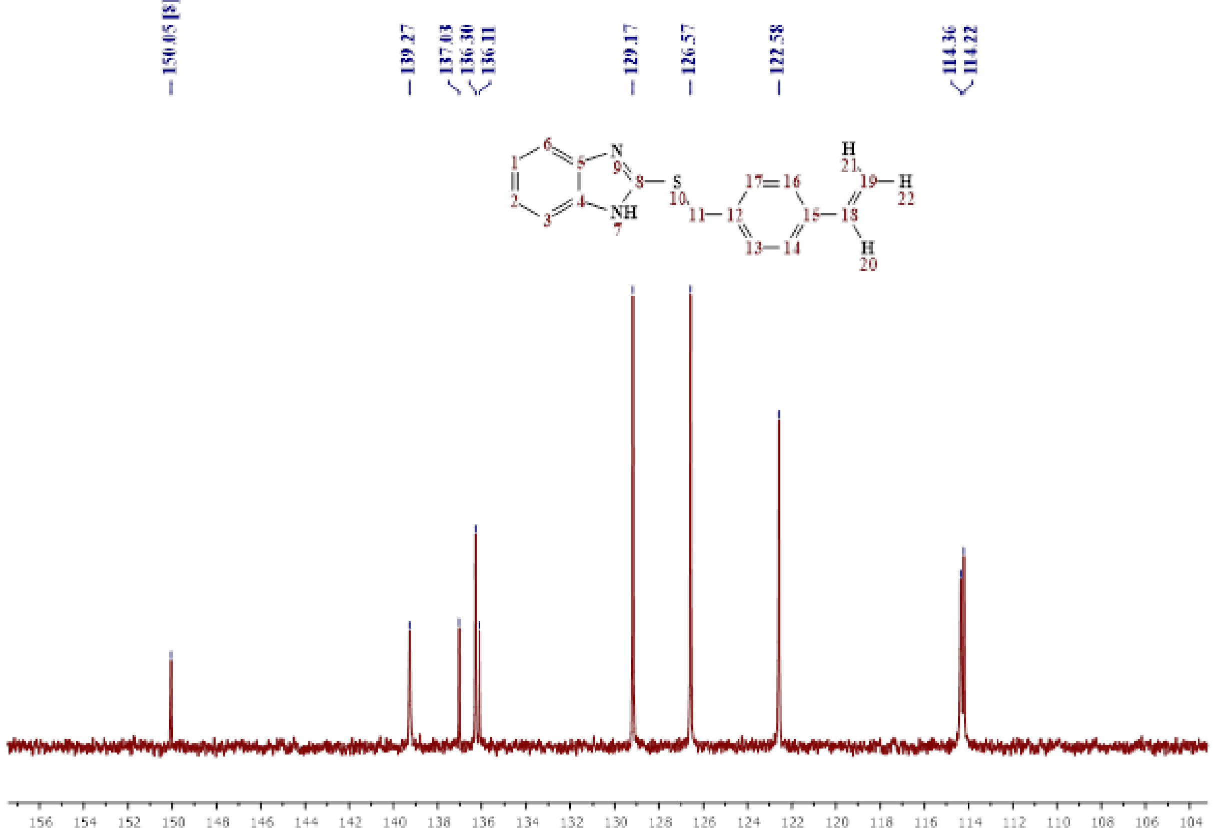

Figure S5. $^{13}C\{^{1}H\}$ NMR (75 MHz, $CDCl_3$) Spectrum for **BZM-2**.

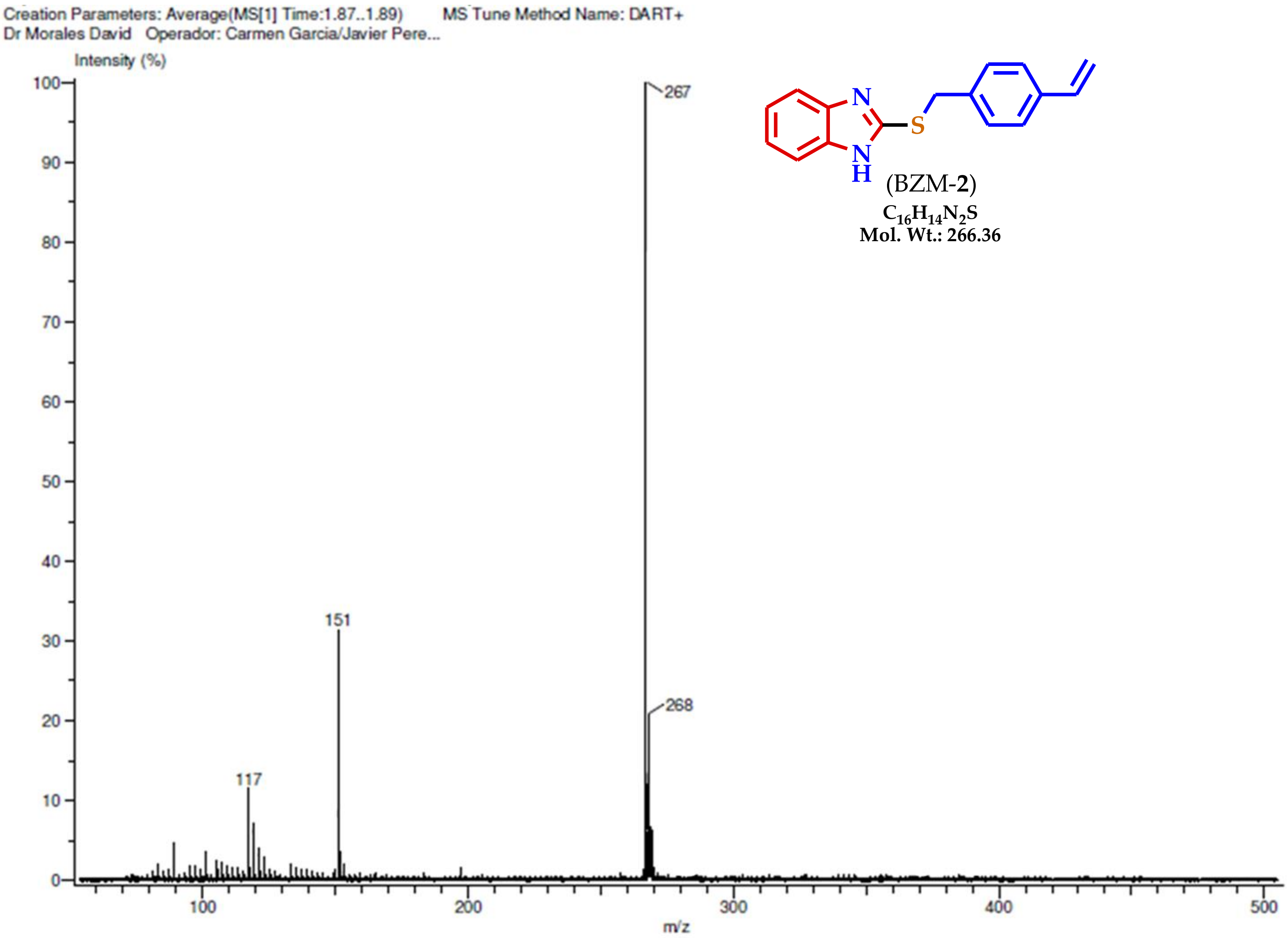

(BZM-**2**)
$C_{16}H_{14}N_2S$
Mol. Wt.: 266.36

Figure S6: Mass spectrometry (DART$^+$) for **BZM-2.**

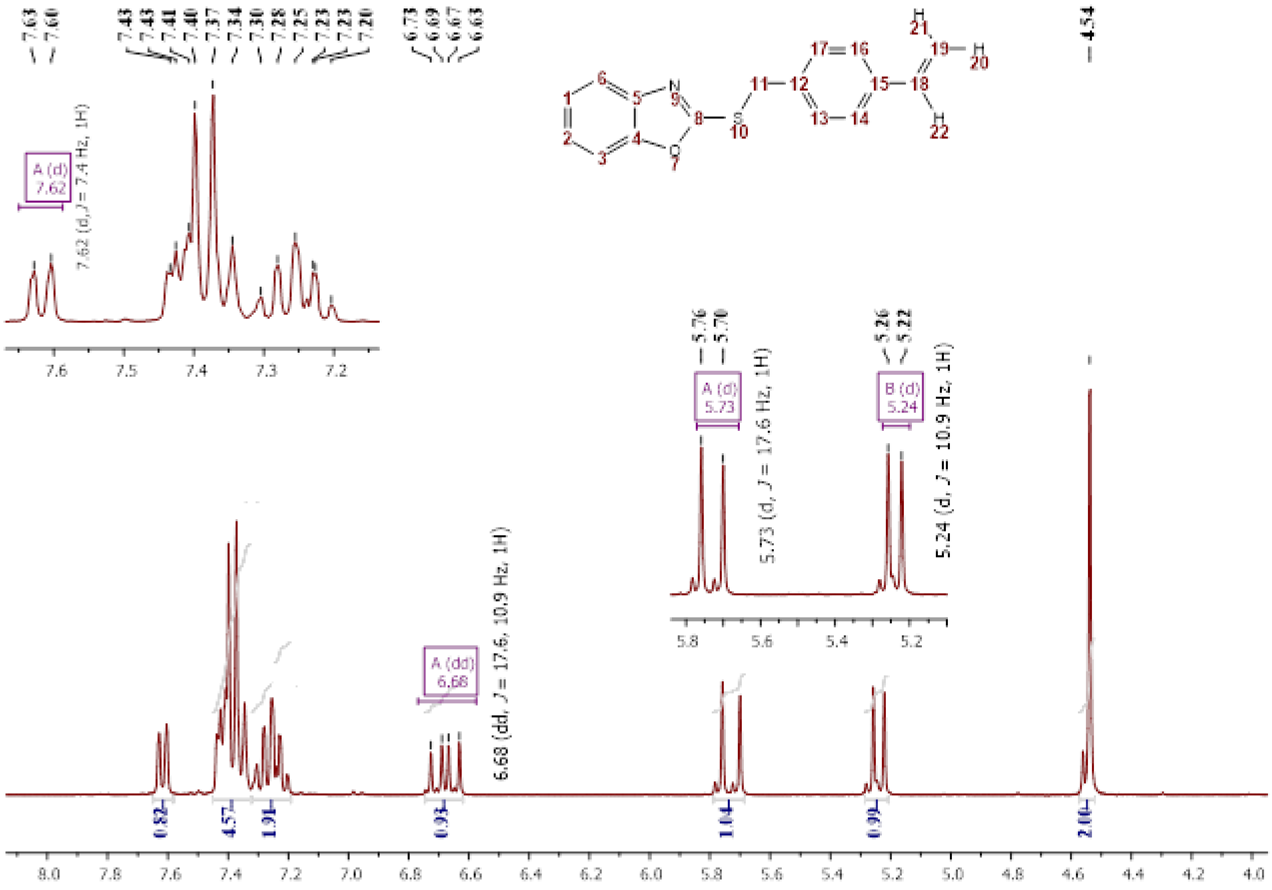

Figure S7. ^{1}H NMR (300 MHz, $CDCl_3$) Spectrum for **BOX-3**.

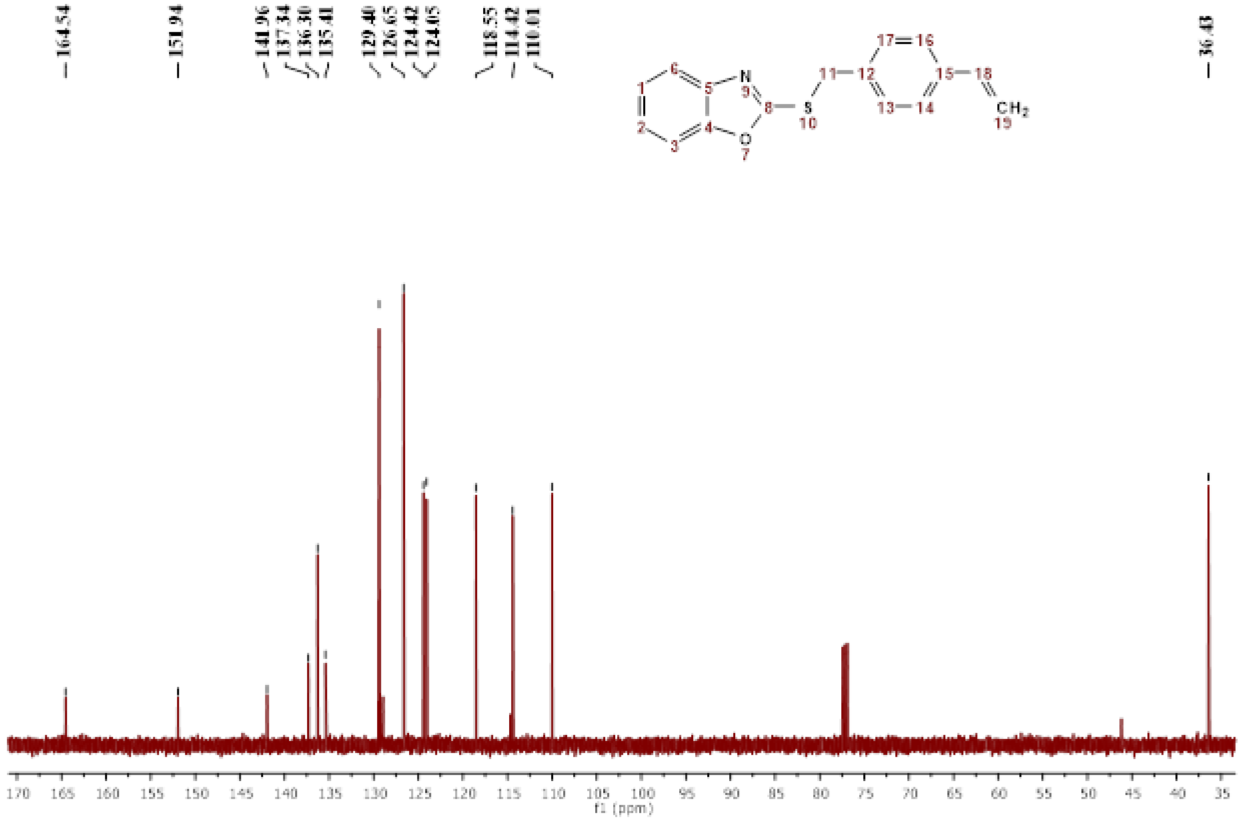

Figure S8. $^{13}C\{^{1}H\}$ NMR (75 MHz, $CDCl_3$) Spectrum for **BOX-3**.

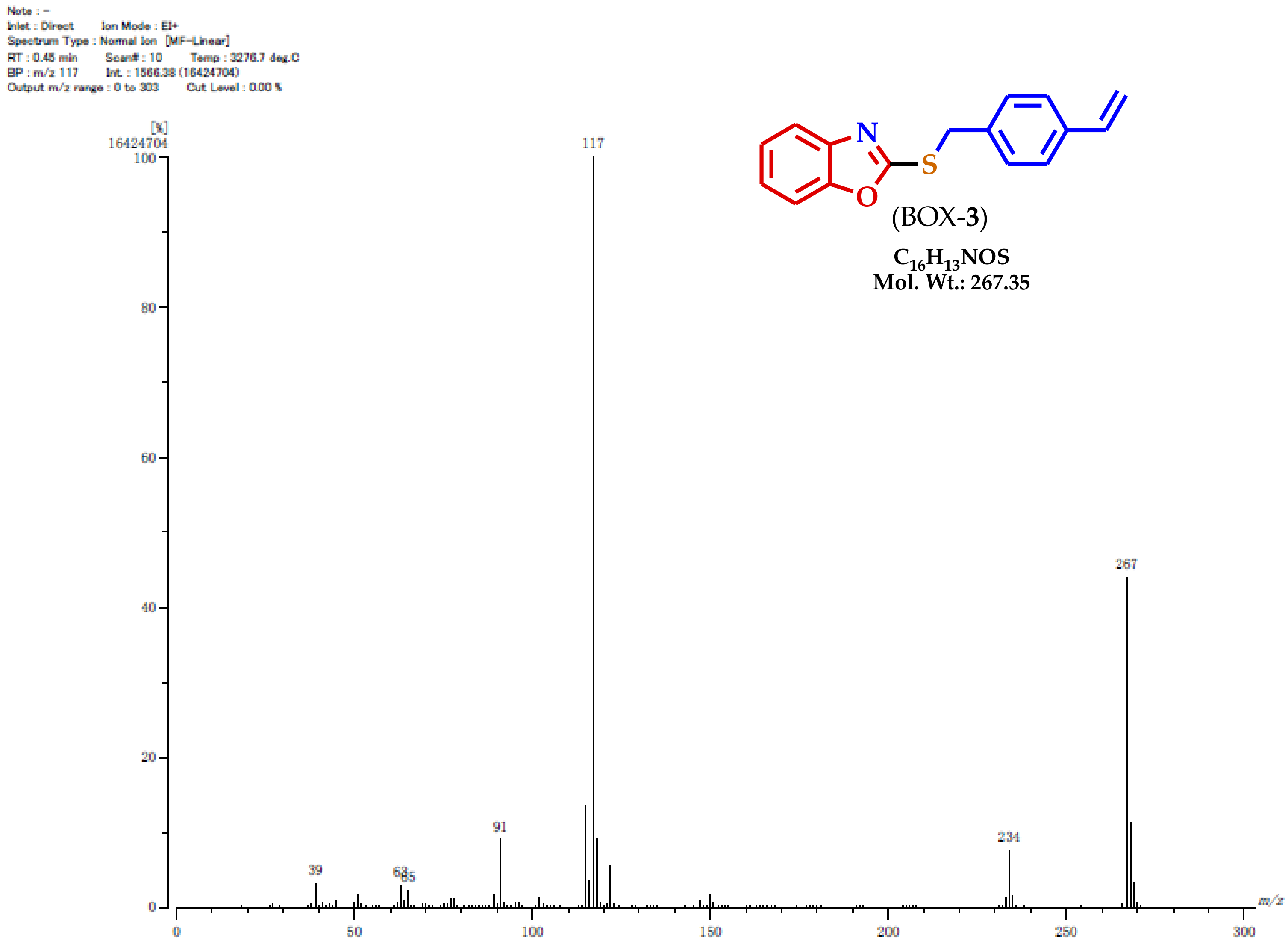

(BOX-**3**)

$C_{16}H_{13}NOS$
Mol. Wt.: 267.35

Figure S9: Mass spectrometry (EI) for **BOX-3.**

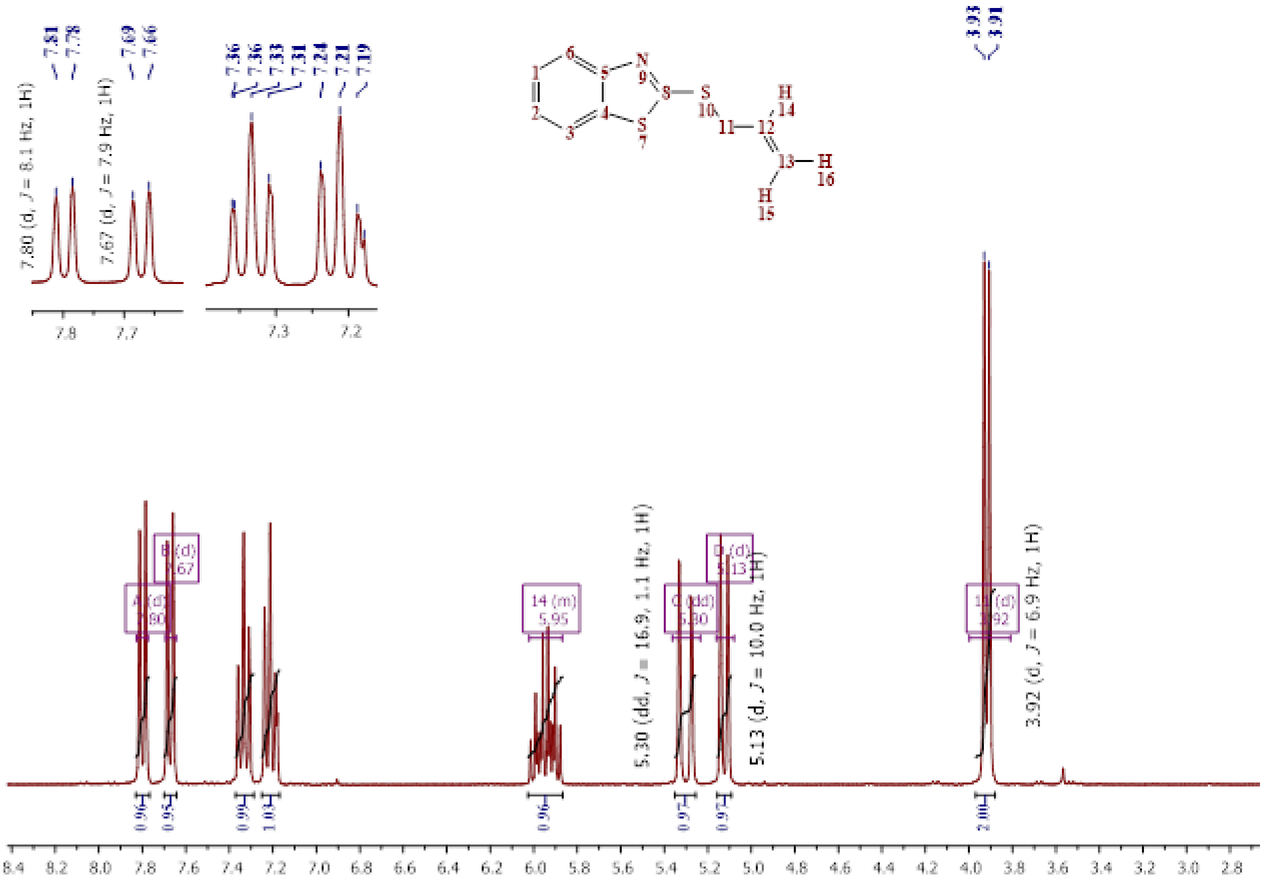

Figure S10. ^{1}H NMR (300 MHz, $CDCl_3$) Spectrum for **BTA-4**.

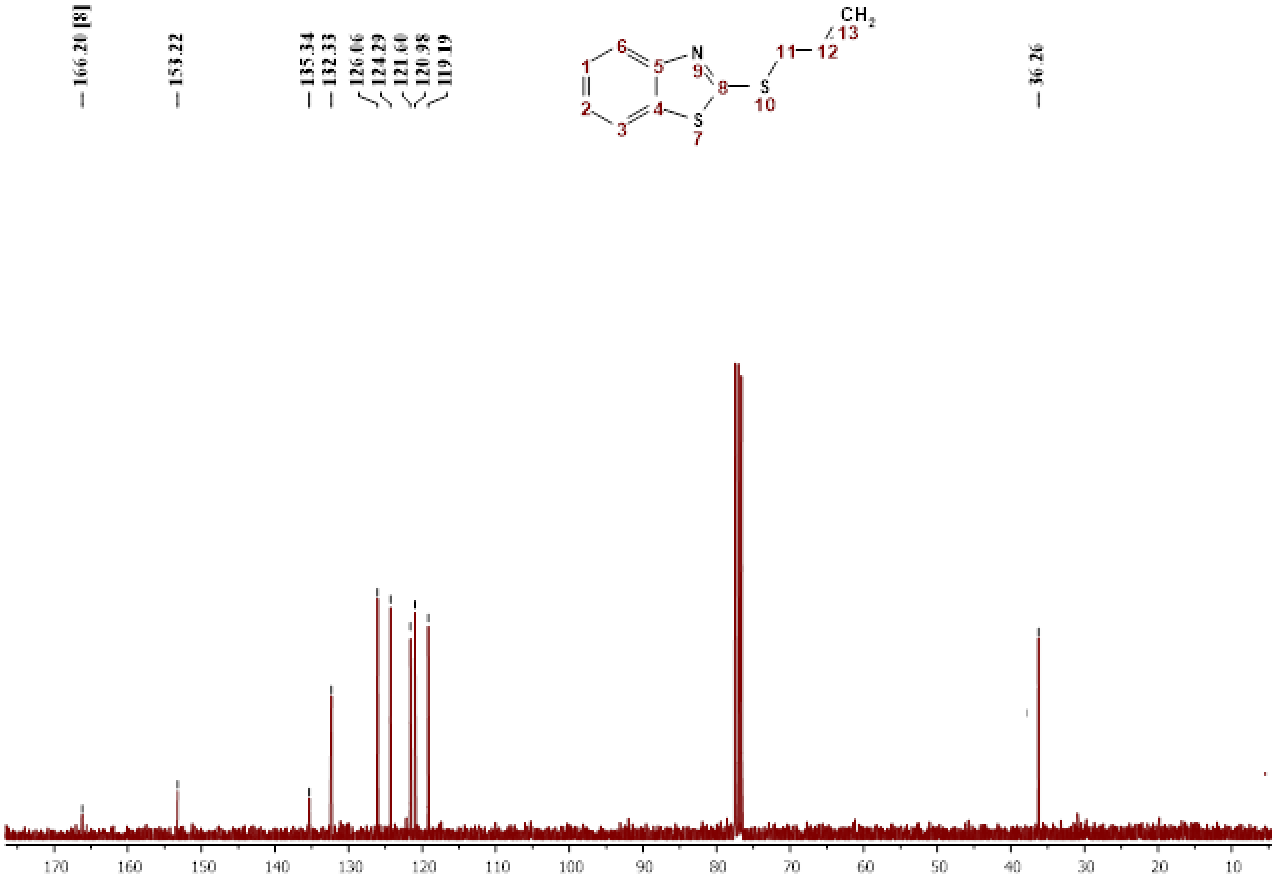

Figure S11. $^{13}C\{^{1}H\}$ NMR (75 MHz, $CDCl_3$) Spectrum for **BTA-4**.

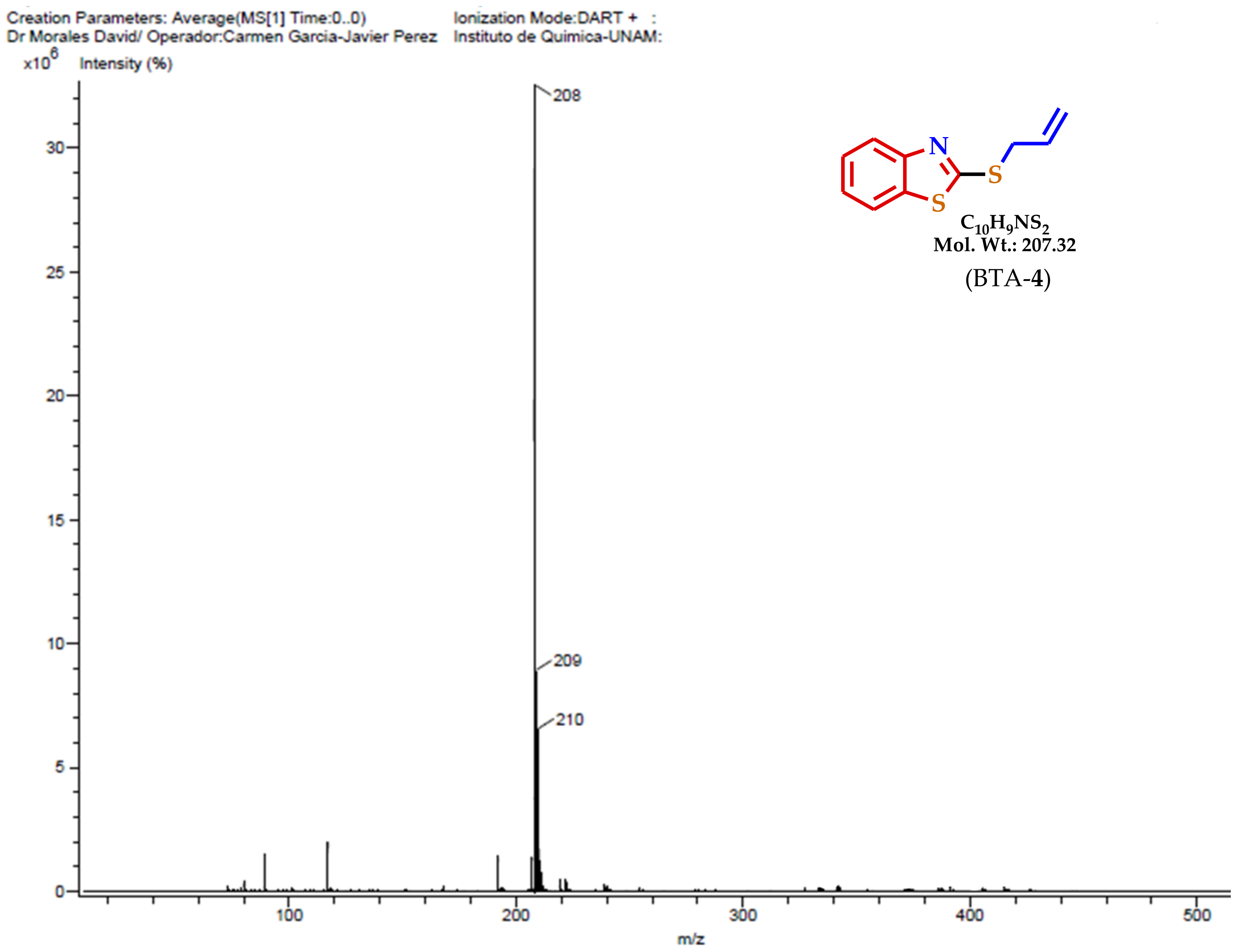

Figure S12: Mass spectrometry (DART⁺) for **BTA-4.**

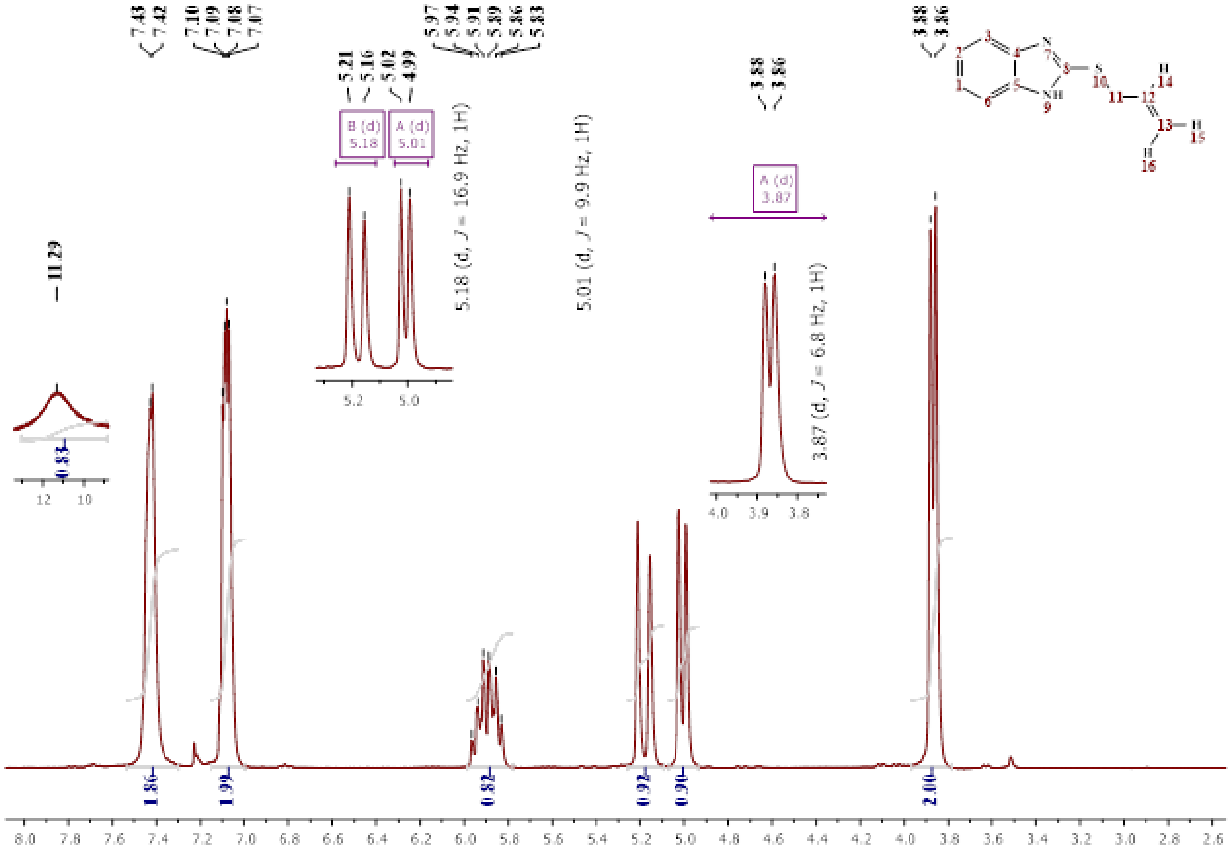

Figure S13. ^{1}H NMR (300 MHz, $CDCl_3$) Spectrum for **BZM-5**.

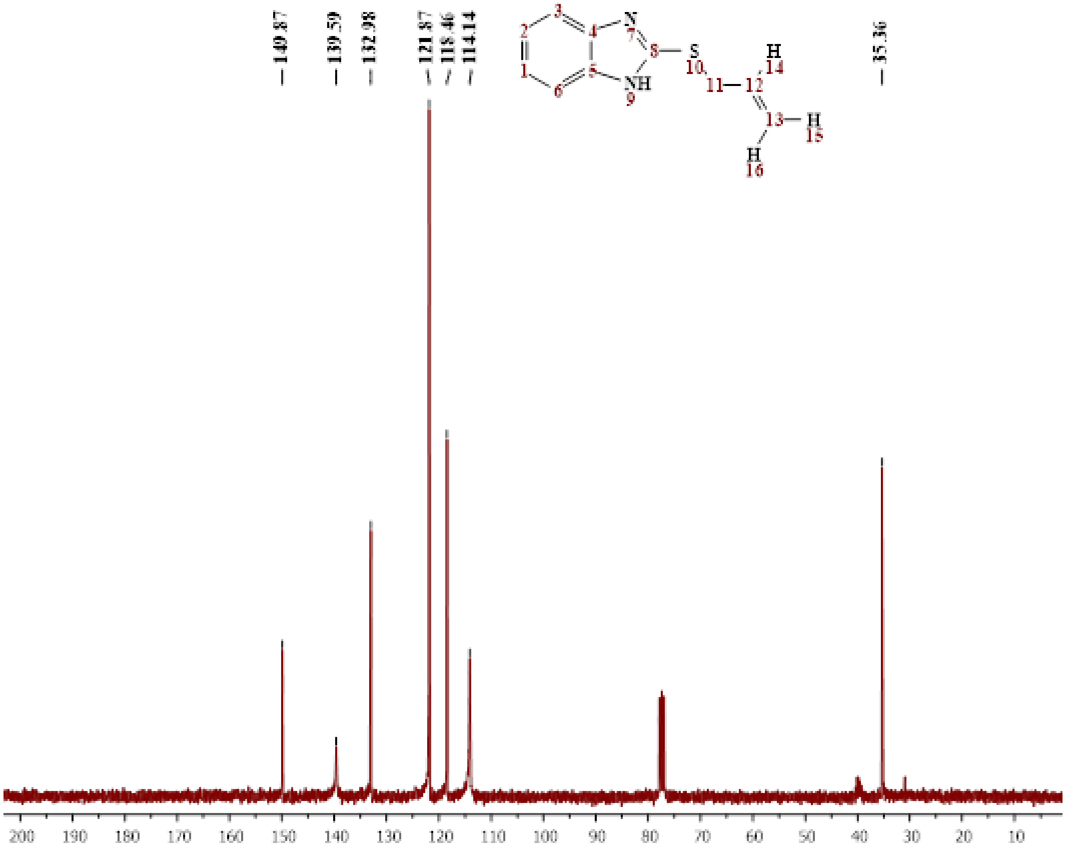

Figure S14. $^{13}C\{^{1}H\}$ NMR (75 MHz, $CDCl_3$) Spectrum for **BZM-5**.

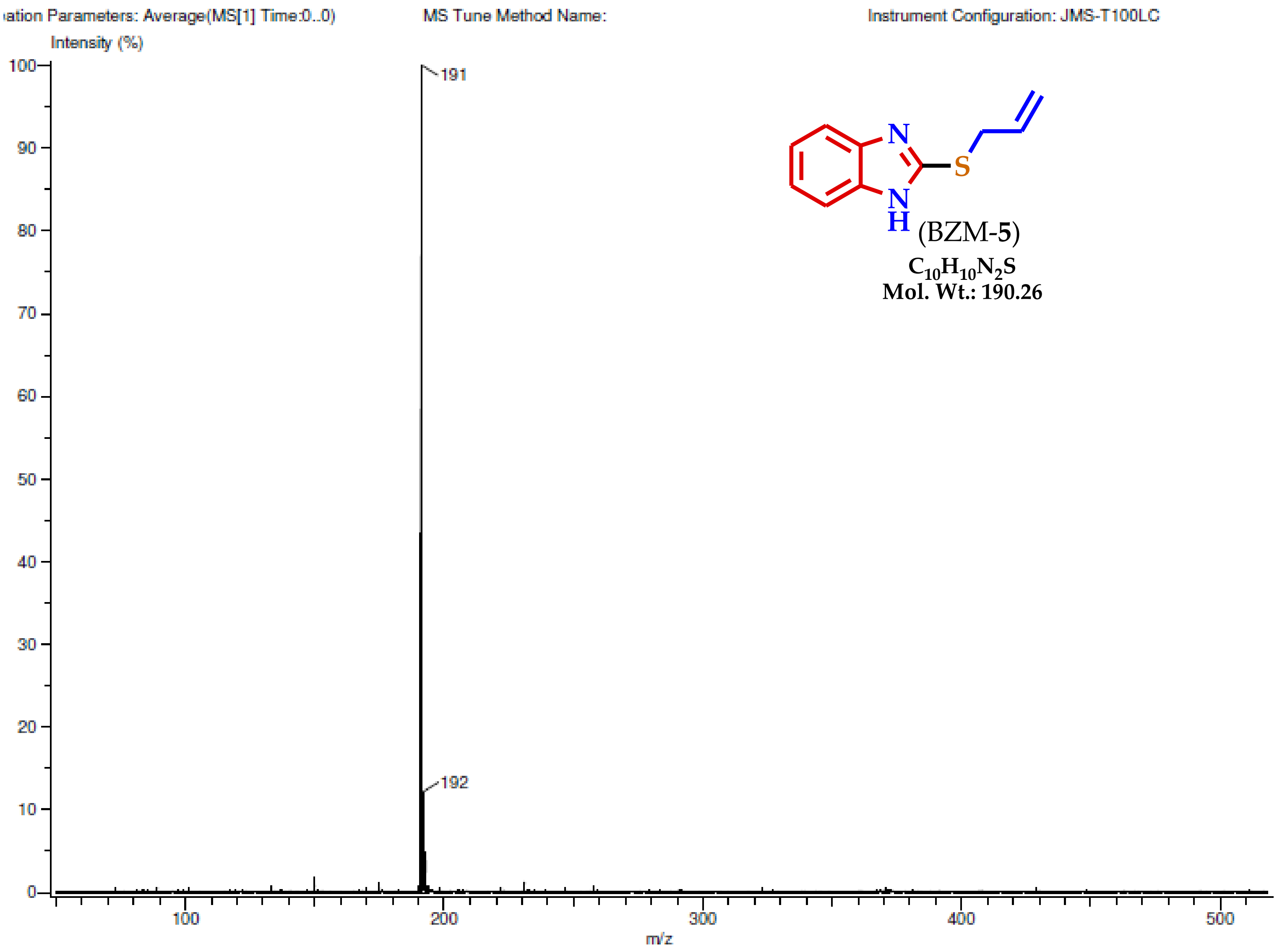

Figure S15: Mass spectrometry (DART$^+$) for **BZM-5.**

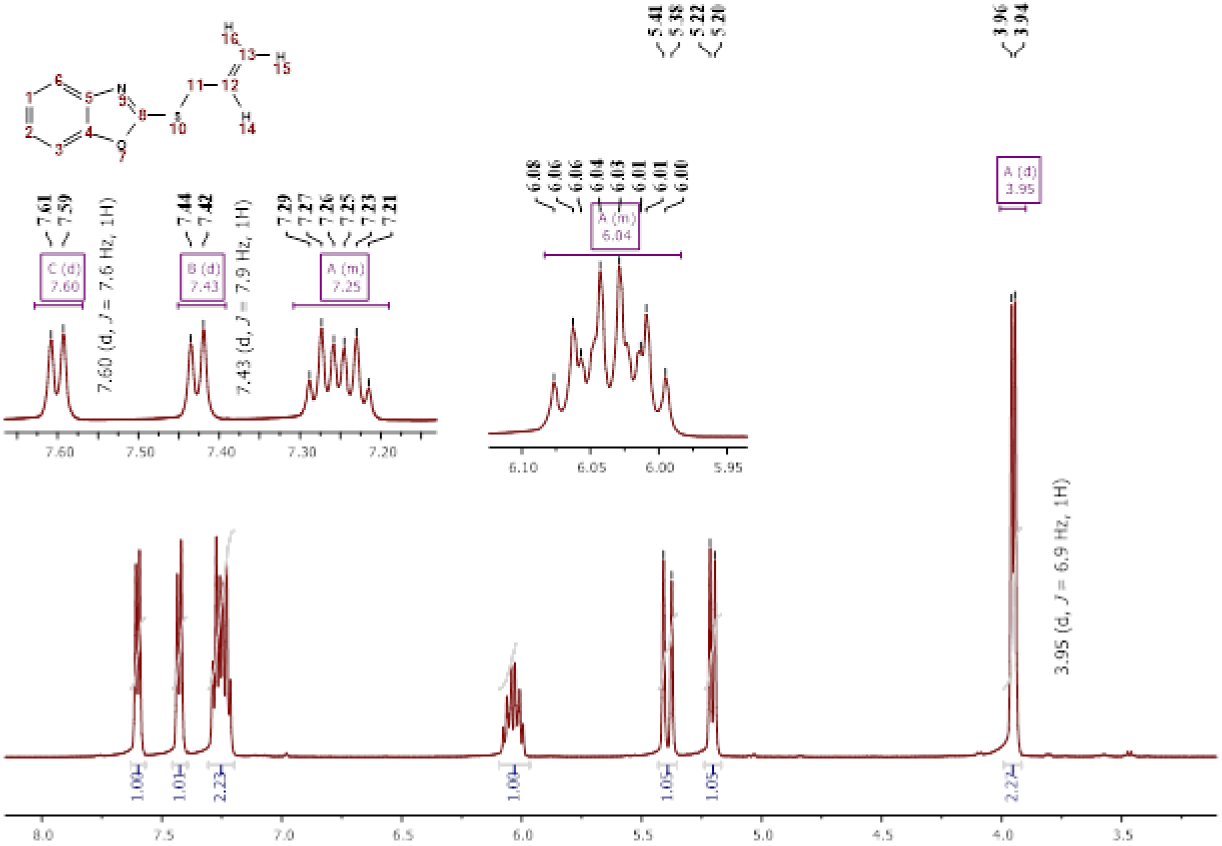

Figure S16. ^{1}H NMR (300 MHz, $CDCl_3$) Spectrum for **BOX-6**.

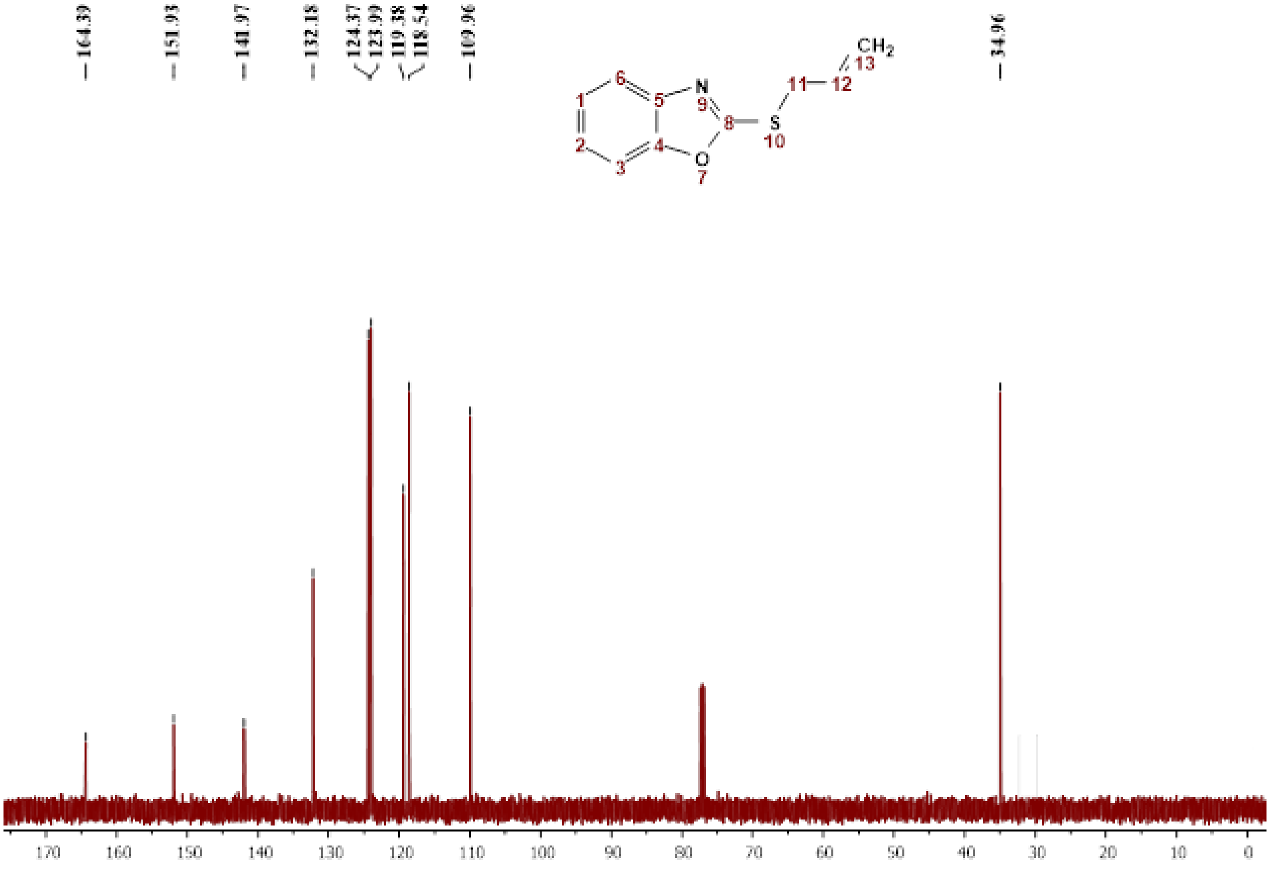

Figure S17. $^{13}C\{^{1}H\}$ NMR (75 MHz, $CDCl_3$) Spectrum for **BOX-6**.

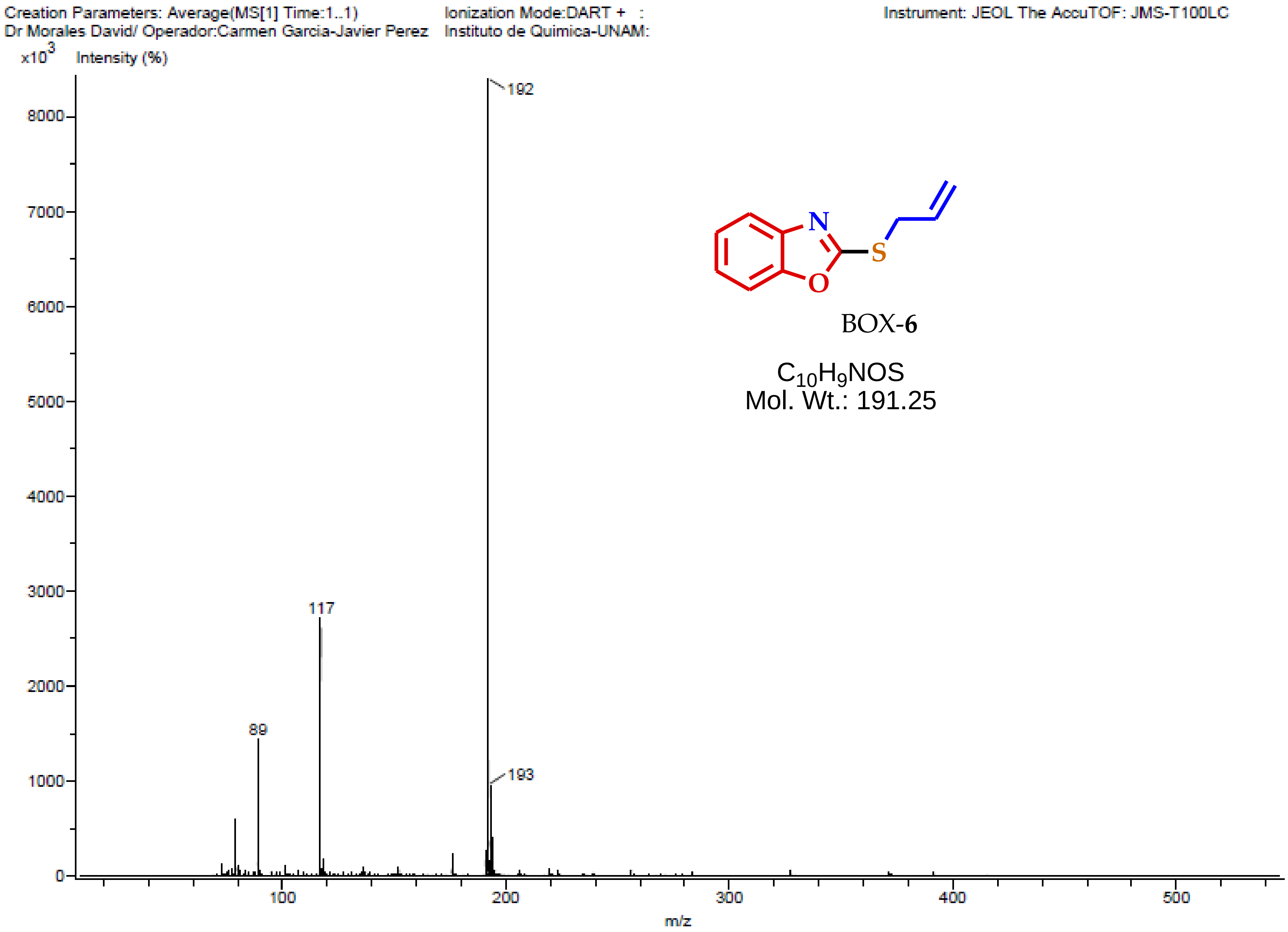

Figure S18: Mass spectrometry (DART+) for **BOX-6**

Table S1. Docking results of receptor EGFR with crystalized ligand as reference and tamoxifen**.**

	EGFR				
	AD4	Vina	Smina	Rank	Position
BTA-1	-5.6	-7.2	-7.4	1.13	3
BZM-2	-5.1	-7.2	-7.4	1.11	4
BOX-3	-5.4	-7.2	-6.8	1.09	5
BTA-4	-3.5	-5.6	-6.2	0.90	6
BZM-5	-3.1	-5.5	-6.3	0.89	7
BOX-6	-3.2	-5.5	-6.1	0.88	8
TAM*	-7.5	-8.1	-6.8	1.25	1
Ref	-6.7	-8.3	-6.7	1.21	2

*Tamoxifen was the crystalized ligand**.**

Table S2. Docking result of receptor Erα with crystalized ligand as reference and tamoxifen.

	Erα				
	AD4	Vina	Smina	Rank	Position
BTA-1	-6.0	-5.6	-6.1	1.00	4
BZM-2	-5.7	-5.9	-7.5	1.07	2
BOX-3	-6.1	-6.4	-5.9	1.03	3
BTA-4	-4.0	-5.2	-6.0	0.89	5
BZM-5	-3.7	-5.4	-6.0	0.89	5
BOX-6	-3.6	-5.0	-6.0	0.87	6
TAM*	-8.1	-6.2	-7.9	1.24	1
Ref	-8.1	-6.2	-7.9	1.24	1

Table S3. Docking result of receptor Pr with crystalized ligand as reference and Tamoxifen.

	Pr				
	AD4	Vina	Smina	Rank	Position
BTA-1	-7.6	-7.3	-7.5	1.24	5
BZM-2	-7.1	-8.3	-7.6	1.28	4
BOX-3	-7.1	-8.4	-7.7	1.30	3
BTA-4	-5.0	-6.3	-6.3	0.99	6
BZM-5	-4.6	-6.3	-6.3	0.98	7
BOX-6	-4.5	-6.4	-6.3	0.98	7
TAM	-8.2	-6.9	-6.9	1.23	2
Ref	-9.4	-8.5	-10.6	1.68	1

Table S4. Docking result of receptor mTOR with crystalized ligand as reference and Tamoxifen.

	mTOR				
	AD4	Vina	Smina	Rank	Position
BTA-1	-6.1	-8.5	-8.9	1.35	4
BZM-2	-5.5	-8.7	-9.4	1.36	3
BOX-3	-6.0	-8.4	-9.4	1.38	5
BTA-4	-3.7	-6.4	-7.6	1.02	6
BZM-5	-3.4	-7.0	-8.1	1.07	7
BOX-6	-3.6	-6.9	-8.0	1.07	7

TAM	-8.9	-8.8	-9.6	1.57	2
Ref	-15.0	-9.1	-6.9	2.12	1

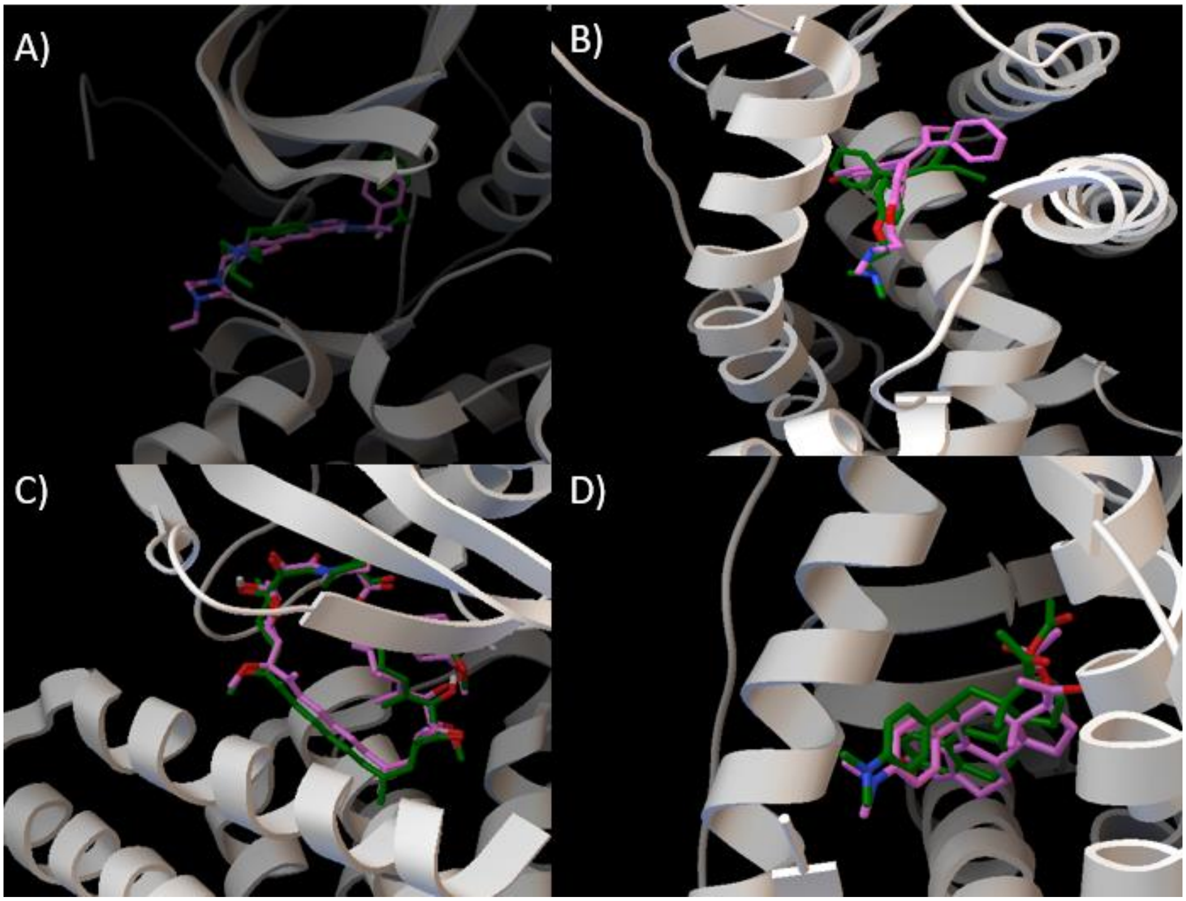

Figure S19. Redocking of crystalized ligand in A) EGFR receptor B) Erα receptor C) mTOR receptor D) Pr Receptor
